# Gratitude enhances widespread dynamic cooperation and inter-brain synchronization in females

**DOI:** 10.1093/scan/nsaf023

**Published:** 2025-03-17

**Authors:** Yangzhuo Li, Xinyu Cheng, Wanqiu Na, Junlong Luo, Xianchun Li

**Affiliations:** School of Psychology, Shanghai Normal University, Shanghai 200234, China; Lab for Educational Big Data and Policymaking, Ministry of Education, Shanghai Normal University, Shanghai 200234, China; Shanghai Key Laboratory of Mental Health and Psychological Crisis Intervention, Affiliated Mental Health Center (ECNU), School of Psychology and Cognitive Science, East China Normal University, Shanghai 200062, China; Institute of Wisdom in China, East China Normal University, Shanghai 200062, China; School of Psychology, Shanghai Normal University, Shanghai 200234, China; Lab for Educational Big Data and Policymaking, Ministry of Education, Shanghai Normal University, Shanghai 200234, China; Shanghai Key Laboratory of Psychotic Disorders, Shanghai Mental Health Center, Shanghai Jiaotong University School of Medicine, Shanghai 200030, China; Department of psychogeriatrics, Third People’s Hospital of Huzhou,Huzhou 313000, China

**Keywords:** gratitude, cost and cost-less cooperation, functional near-infrared spectroscopy (fNIRS), inter-brain synchronization (IBS), dynamics

## Abstract

From both social and life perspectives, gratitude is essential for managing social relationships and fostering cooperation. This study investigated the dynamic influence of gratitude on two modalities of cooperative interactions and the associated interpersonal dynamic neural mechanism by integrating a dyadic ecological paradigm with functional near-infrared spectroscopy). Several critical findings emerged: the gratitude group exhibited better cooperative behaviors compared to the joy and neutral groups in both the Prisoner’s Dilemma Game (PDG) and the Button-press Game (BPG). The dynamic cooperative behaviors further elucidated that gratitude dynamically facilitates cooperation by boosting inclusivity towards the benefactor’s slight defect in the PDG and heightening action coordination in the BPG. Accordingly, higher inter-brain synchronization (IBS) was predominantly observed in the left and right middle frontal gyrus and the right sensorimotor cortex in the gratitude, compared to the joy and neutral groups. Moreover, the gratitude group exhibited increased IBS over time (across blocks) in the left and right middle frontal gyrus and the right superior temporal gyrus. These findings substantiate that gratitude facilitates widespread social cooperation and progressively enhances IBS among individuals. This work advances our understanding of gratitude-induced large-scale cooperative behaviors in societies.

## Introduction

Gratitude, an essential element of human social–moral emotion, stems from the perception of benefiting from the kindness of others (McCullough et al. 2001, Algoe et al. 2008). From a functional or adaptationist perspective, gratitude is believed to have evolved to serve adaptive functions, especially in motivating individuals to reciprocate moral behaviors and solve challenges in cooperative interactions (Algoe 2012, Yu et al. 2017, Yost-Dubrow and Dunham 2018, DeSteno et al. 2019, Sznycer and Lukaszewski 2019, Tsang and Martin 2019). Therefore, beyond being an emotional experience, gratitude is recognized as the “parent of other virtues” and a “moral barometer” in responding to kindness, influencing cooperative tendencies and behaviors (McCullough et al. 2001). Prior research has highlighted the role of gratitude in solidifying cooperation and elucidating the neurocognitive mechanisms involved (Bartlett and DeSteno 2006, Tsang 2006, DeSteno et al. 2010, Rumble et al. 2010). Despite the insights from these pioneering works, the literature still presents numerous unresolved issues. For gratitude to assume pivotal role in enhancing and sustaining cooperation-based relationships, it must extend beyond simple reciprocation of kind acts between individuals (Algoe et al. 2008, Algoe 2012, Zhu et al. 2021). Thus, gratitude must serve as an effective mechanism that widely motivates cooperative behaviors. However, existing research often explores the effects of gratitude in isolated setups, a systematic exploration of gratitude’s impact on diverse cooperative behaviors, particularly in spontaneous and interactive contexts, remains inadequate.

Here, we concentrate on two well-established modes of cooperation: cooperation in situations involving cost or costless. The Prisoner’s Dilemma Game (PDG) provides a framework for examining whether and when individuals decide to cooperate in costly situations (Tang et al. 2016, Barclay et al. 2021). Specifically, two players decide whether to “cooperate” or “defect” (i.e. not cooperate) and receive a payoff based on their joint outcomes. The PDG task retains a dominant strategy of defection for purely selfish agents (St-Pierre et al. 2009, Tang et al. 2016, Barclay et al. 2021). The temptation to defect arises from the potential personal benefit, whereas cooperation requires foregoing a potentially better outcome for oneself. Hence, cooperation in the PDG is efficient but costly. Conversely, the Button-press Game (BPG) provides a formal framework to examine cooperation in a costless situation (Li et al. 2022, Liu et al. 2024). The BPG is an action-consistent cooperation task, analogous to real-life consistent action settings such as synchronized marching in the military. In this task, two players press buttons simultaneously, coordinating their rhythms to accomplish a common goal (similar as Cui et al. 2012). In this scenario, two players are equal and face no temptation to defect, that is, the so-called “sharing weal and woe” (Liu et al. 2024).

Furthermore, these two forms of cooperation exhibit shared characteristics. From an evolutionary perspective, both are recognized as social patterns essential for survival, albeit through distinct pathways (Molleman et al. 2019, Liu et al. 2024). Additionally, both forms require cognitive effort such as monitoring and mentalizing abilities to attribute independent mental states, including thoughts, beliefs, and desires, thereby facilitating union with others (Cui et al. 2012, Tang et al. 2016, Fehr and Schurtenberger 2018). These cognitive processes also allow individuals to anticipate and predict others’ intentions and actions, and thus adapt their behavior accordingly (Barclay et al. 2021, Liu et al. 2024). Given these considerations, these two modalities of cooperation can serve as a solid model to illuminate the social significance of gratitude in cooperative engagements.

Notably, significant cooperative interactions are seldom single-shot, and gratitude can dynamically shape individuals as they interact over time (Cochard et al. 2004, Bartlett et al. 2012). Traditional research has predominantly focused on static cooperation, neglecting the dynamic interpersonal interactions that could limit the exploration of gratitude-induced behavioral adjustments during cooperation. Grateful individuals, as an endogenous motivational state, consider the potential social relationships that aid in facilitating mutual understanding and anticipation, thus improving interactions through constantly updated cooperative experiences (Algoe 2012, Fareri et al. 2015). Moreover, as gratitude has evolved beyond simple reciprocity, it inspires individuals or groups to engage in relationship maintenance, such as offering to do more than what is obligated (Kubacka et al. 2011). Evidence is accumulating that feeling grateful facilitates the cognitive inhibition of self-centered impulses (Zhu et al. 2020, Dickens and DeSteno 2016), encourages reciprocation with costly helping at the expense of individual gain (Bartlett and DeSteno 2006, Tsang 2006, DeSteno et al. 2010), and reinforces socially inclusive behaviors towards benefactors (Bartlett et al. 2012, Zhu et al. 2021). Collectively, a comprehensive understanding of gratitude necessitates a broad framework that captures both initial and evolving social cooperation interactions.

Given the interactive and dynamic nature of cooperation, it was imperative to adopt the “social brain” technique, which measures brain activity from two or more individuals simultaneously (Czeszumski et al. 2020, Lieberz et al. 2021). Inter-brain synchronization (IBS)—the neurophysiological substrates of “social brain”—is postulated as an evolutionary adaptive mechanism for social communication and interaction (Holroyd 2022; Müller et al. 2021, Shamay-Tsoory and Mendelsohn 2019). IBS captures dynamic features unique to social interactions (Jiang et al. 2015, Li et al. 2022, Pan et al. 2023). Numerous studies have offered supportive evidence that greater IBS in the superior frontal cortex (SFC), middle frontal cortex (MFC), frontopolar cortex (FPC), superior temporal sulcus (STS), and right temporo-parietal junction (rTPJ) during various cooperative interactions positively predicts cooperative levels across dyads and groups (Cui et al. 2012, Li et al. 2022, Liu et al. 2021). Therefore, this study focused on the IBS mechanisms underlying gratitude-induced cooperative interactions. Functional near-infrared spectroscopy (fNIRS) was selected for its mobility, relatively low cost, suitable temporal and spatial resolution (Yücel et al. 2021). The prefrontal and right temporoparietal cortices were chosen as priori areas of interest in this study, as these regions are crucial for the neural correlates of IBS in cooperative interactions (Cui et al. 2012, Tang et al. 2016; Li et al. 2022). Additionally, these brain regions also play roles in emotional processes, affective consideration and the effects of gratitude (Van Overwalle 2011, Balconi et al. 2020).

In light of the above, it remains unclear the dynamic influence of gratitude on various modalities of cooperative behavior, and the underlying dynamic inter-neural basis awaits elucidation. Along these lines, the present study utilized a modified two-round resource distribution task (RDT, Tsang 2006) to induce gratitude. We also introduced joy and neutral to eliminate any confounding effects of emotional valence (Sasaki et al. 2020). For the cost cooperation situation, we integrated a revised two-person multi-round PDG task. To avoid any potential biases, participants played the games anonymously in each session (i.e. block). For the costless cooperation situation, we employed a two-person multi-round BPG task in accordance with Cui et al. (2012). Each task was divided into two sessions (blocks) to examine the temporal dynamics of cooperation. Additionally, participants were prompted to report their psychological feelings to investigate potential psychological contributors to gratitude-induced differences in cooperative behaviors.

## Methods

### Participants and ethics

A total of 186 female adults [93 dyads, aged 21.78 ± 2.19 years (mean ± s.d.)] were involved as paid volunteers. Only females were recruited to control for potential gender differences, as gender-based pairings have a well-documented influence on cooperation (Cheng et al. 2015, Baker et al. 2016). Additionally, females are more proficient in expressing emotions and providing emotional support (Drążkowski et al. 2017). Members of each dyad were strangers to avoid potential confounds of familiarity (Baker et al. 2016, Liu et al. 2024). All participants were right-handed (Oldfield 1971), with normal or corrected-to-normal vision, and reported no history of neurological or psychiatric disorders. None of the participants were using psychiatric medication or legally prohibited drugs, and none were currently pregnant or breastfeeding. Participant dyads were randomly assigned to one of three emotion groups: Gratitude, Joy, or Neutral. Ten dyads were excluded due to misunderstandings regarding the experimental requirements or failure in fNIRS recording, resulting in a sample size of 83 dyads [30 in Gratitude, 27 in Joy and 26 in Neutral; aged 21.04 ± 2.16 years (mean ± s.d.), matched across the three groups, see Supplementary Table 1]. A post-hoc power analysis using G*power 3.1 software indicated that the sample size of 83 dyads would be sufficiently powerful (1 − *β* = 0.92) at a significance alpha level of α = 0.05.

The experimental protocol was approved by the Human Research Protection Committee at East China Normal University (Number HR 032-2021). All procedures were thoroughly explained to the participants and were performed in accordance with the ethical guidelines. Participants gave written informed consent and were free to leave the experiment at any time.

### Experimental section

#### Procedure.

**Figure 1.**
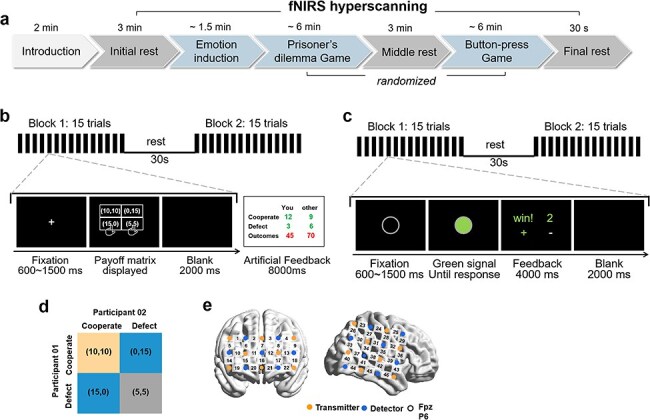
Experimental protocol. (a) Schematic of the experimental protocol. (b) PDG design, composing of 2 blocks of 15 trials each, with 1 artificial outcome feedback after Block1. (c) BPG design, composing of 2 blocks of 15 trials each. The sequence of two tasks (PDG and BPG) was counterbalanced across dyads. (d) Payoff matrix of the PDG. The duration of the payoff matrix display period was not fixed, with participants taking ∼3–8 s to make their choices. (e) fNIRS probe positions. The transmitter and detector were located in the prefrontal and right temporoparietal regions, respectively, with an inter-optode distance of 30 mm. The frames that connect the sources and detectors and the numbers shown in black represent the measurement channels (CH). 3 × 5: the middle optode of the lowest probe row of the patch was placed at Fpz; 4 × 4: the left detector of the third row coincided with P6, and the patch was placed horizontally, following the international 10–20 system. All optode probe sets were positioned using individualized caps made from swimming caps, ensuring signal consistency across variations in head size.

Two participants were concurrently invited into the lab and equipped with both oral and written explanations of the experimental procedure. All emotion groups shared similar procedure except for the emotion inductions applied. Before the formal experiment started, all participants furnished sociodemographic information (such as age and education level) and pre-task emotion scale (e.g. *Positive and Negative Affect Schedule*, Watson et al. 1988). No significant differences were observed among the three emotion groups regarding these variables (Supplementary Table S1). The experiment timeline is illustrated in Fig. 1a. After an initial rest (3 min), a two-round resource distribution task (RDT) modified from Tsang (2006) was carried out to elicit the target emotion (Gratitude, Joy, or Neutral). Subsequently, participant dyads engaged in two computer-based cooperative interaction tasks, comprising a modified PDG (Fig. 1b and d) and a BPG(Fig. 1c). Each cooperation task comprised two blocks of 15 trials each, with a 30 s rest period between blocks. The order of the two cooperative interaction tasks was randomized across dyads, with a 3-min rest period between tasks to mitigate carry-over effects. Participants were not notified of the exact number of trials to prevent adjustments to their response strategies based on anticipated trial counts. Concurrently, fNIRS was recorded from the dyads throughout the experiment.

Participants were instructed to sit still, close their eyes, refrain from moving, and relax their minds during the rest periods (Jiang et al. 2015, Zhu et al. 2021). Each participant in the dyad faced one of two computer screens displaying the task, and each used one of two keyboards to record their behavioral responses. Two members of the dyad sat across the table, with a baffle in the middle to prevent visual contact during the experiment. All participants wore headphones to mask keypress sounds and to preclude any verbal or nonverbal communication between them. The task materials are detailed in the succeeding section.

#### Emotion induction.

Tsang (2006). We developed a novel two-round RDT paradigm to manipulate emotion, inspired by Tsang (2006). In this task, two participants simultaneously received coin allocations from either a partner or a computer, randomly determined in each round. Specifically, participants were informed to allocate 10 yuan in each round, with the divider randomly selected by one of them or the computer. If the divider was one member of the dyad, they had the opportunity to let a message for the other. However, all allocations were predetermined, and both dyad members viewed identical information simultaneously. In Round 1, participants received 3 yuan by chance in the Gratitude and Joy groups while being informed that their partner received 7 yuan; participants each received 5 yuan in the Neutral group. In Round 2, the divider in the Gratitude was identified as “their partner” (another participant), who allocated 7 yuan to them and displayed a message on the screen: “I saw you didn’t get much in the last round—that must’ve been a bummer, so I gave a little more in this round”. In contrast, in the Joy and Neutral groups, the computer served as the divider. Participants received 7 yuan by chance in the Joy group, while they received 5 yuan (same as Round 1) in the Neutral group.

Before (baseline) and after each round (Rond1 and Round2), participants completed a general positivity rating based on their mean response to three items: “How happy/amused/pleasant do you feel?” using a 7-point scale (1= not at all, 7 = very much). They also reported whether they felt grateful or thankful towards their partner (“Yes”/“No”) to examine the validity of the emotion manipulation.

#### Prisoner’s Dilemma game.

The PDG task was adapted to evaluate cooperation in a costly situation (e.g. Tang et al. 2016). As indicated in the payoff matrix in Fig. 1d, if both participants chose to cooperate, they each earned 10 yuan; if both chose to defect, they each only earned 5 yuan; if one cooperated while the other defected, the defector received 15 yuan while the cooperator received nothing. Each participant made their decisions privately in each round. As with most PDG studies, participants could potentially gain more by choosing to defect rather than cooperate; however, mutual defection would result in a greater loss (Bone et al. 2015, Barclay et al. 2021). Accordingly, there were three possible outcomes: both participants cooperate (CC), one cooperates while the other defects (CD), or both defect (DD). It was emphasized that participants would receive the average amount of their earnings from the entire game as a reward, encouraging them to maximize their earnings. Four practice trials were conducted before the formal experiment to ensure that participants understood the payoff matrix.

To ensure a robust effect of gratitude and to avoid any confounding effects, such as participants’ choices being effected by preceding outcomes, especially their partner’s decision in the earlier trial, participants were asked to decide whether to cooperate or defect without knowing their partner’s decision within a complete block (Acedo and Gomila, 2013). One feedback was available to each participant after Block1 and before Block2, summarizing the cumulative choices and benefits of both members. In fact, we artificially manipulated the partner’s defections to be slightly higher than those of the participants themselves. Specifically, the number of partner defections was always three more than the participant’s defections (i.e. if one cooperated 12 times and defected 3 times in Block1, the feedback would show their partner cooperated 9 times and defected 6 times). The upper limit of defections was set at 15 times. This arrangement aimed to further probe the impact of gratitude on participants’ trade-offs between mutual benefit and defect aversion. We emphasized that the monetary payoffs were real and no deception was used.

To further investigate potential psychological contributors to gratitude-induced differences in the PDG task, participants filled out self-report measures after each block containing five items: (i) “express gratitude,” (ii) “trust the partner,” (iii) “establish justice,” (iv) “to get money,” and (v) “fulfill an obligation,” on a 7-point scale (1 = not at all, 7 = very much) (see Supplementary Table S2).

#### Button-press Game.

In accordance with Cui et al. (2012), participants were instructed to press their buttons as simultaneously as possible in this task. Each trial commenced with a hollow gray circle displayed on the participants’ computer screens for an unpredictable interval between 0.6 and 1.5s. Subsequently, a rapid change in the color of the circle from gray to green initiated a button press response from both participants in dyad using their right index or middle finger. If the latency between their button presses was below a threshold [*T* = (RT1 + RT2)/8, where RT1 and RT2 were the reaction times of the two participants respectively], they would earn one point together; otherwise, the point would not be counted. The parameter 1/8 was chosen to maintain a moderate level of difficulty for the cooperation (Cui et al. 2012; Li et al. 2022). After both participants responded, a feedback screen was displayed for a duration of 4s, showing the result of the current trial (“Win!” Or “Lost!”) along with their cumulative points. The feedback also indicated whether the participant responded faster (green “+”) or slower (white “−”) than their partner, on both participants’ right-hand sides of the monitor. An inter-trial interval (black screen, 2s) was shown after the feedback, followed by the subsequent trial. Four practice trials were conducted before the formal experiment to ensure that participants got the rules.

After completing the BPG task, each member of the dyad was given a post-task questionnaire to assess generalized performance and efficacy using a 5-point Likert scale (1 = not at all, 5 = very much). Evaluations included: (i) satisfaction with their own performance, (ii) satisfaction with their partner’s performance, and (iii) perceived cooperativeness.

### fNIRS data collection

The brain activity of dyads was simultaneously recorded using an ETG-4000 optical topography system (Hitachi Medical Corporation, Japan). Optical data were collected at wavelengths of 695 and 830 nm, with a sampling rate of 10 Hz. Two probe sets were placed over the prefrontal and right temporoparietal regions for each participant. Specifically, a 3 × 5 probe covered the prefrontal area (eight transmitters and seven detectors, yielding 22 measurement channels, i.e. CH1–CH22) and a 4 × 4 probe covered the right temporoparietal area (eight transmitters and eight detectors, yielding 24 measurement channels, i.e. CH23–CH46), see Fig. 1e for the reference positions and channel locations. The probe set locations were checked and adjusted to ensure consistency among the participants within each dyad and across all dyads. A three-dimensional digitizer (Xiao et al. 2017) and NIRS-SPM software (Singh et al. 2005, Ye et al. 2009) were employed to reveal the anatomical locations of the CHs. Specifically, we used the three-dimensional digitizer to obtain the locations of CHs on the participants’ head, and the NIRS-SPM software for MATLAB validated these location data. The anatomical locations and the atlas-based data are listed in Supplementary Table S9.

## Statistical analysis

### Emotion induction manipulation check

A linear mixed-effects model was conducted on the general positivity rating in the RDT, with Round (baseline vs Round1 vs Round2) as a within-subjects factor, Group (Gratitude vs Joy vs Neutral) as a between-subjects factor, and their interaction. Bonferroni correction was used for multiple comparisons (threshold: *P*_bonf_ < .05). To further assess the successful induction of gratitude, a chi-squared test compared the proportion of participants “grateful to their partner” between groups.

### PDG task behavioral and psychological measures analyses

We examined the General cooperation outcomes, calculated as the ratio of CC, CD, and DD in each dyad. A two-way mixed-model ANOVA was applied to the general cooperation outcomes with Groups (Gratitude vs Joy vs Neutral) as the between-subjects factor and Type (CC vs CD vs DD) as the within-subjects factor, using Bonferroni correction for multiple comparisons. Additionally, to evaluate the temporal dynamic effect of cooperation and the impact of feedback, CC, CD, and DD ratios were counted for Block1 and Block2, respectively. We then submitted these metrics to mixed-model ANOVAs with Bonferroni correction. For completeness, we also directly examined the difference between Blocks by paired-sample *t*-tests (Bock1 vs Block2) for each Type in each Group.

Next, we submitted each of the five psychological measures hypothesized to drive the gratitude-induced differences in cooperative performance to a one-way ANOVA, with Group as the between-subjects factor, for both Block1 and Block2. Bonferroni correction was implemented to adjust the *P*-values of the five statistical tests. Rating scores were averaged within the dyads to create a dyad-level variable for analysis. Additionally, we investigated the relationships among psychological measures and cooperative behaviors at the dyad level. Specifically, Pearson correlations were used to identify significant associations between cooperative behaviors and each of the five psychological measures in Block1 and Block2, respectively. Bonferroni correction was utilized to adjust *P*-values.

### BPG task behavioral analyses

Two metrics were utilized to quantify cooperative performance in the BPG: (i) the Effective Adjustment rate and (ii) the Mean Cooperation rate. Consistent with our previous research (Li et al. 2022), the Effective Adjustment rate represents participants’ willingness to adjust their response speed according to the feedback screen to achieve synchronized pressing with their partner. If the participant’s response speed (faster or slower) was consistent with the feedback from the preceding trial (“−” or “+”), the trial was considered an effective adjustment. A higher number of effective adjustments indicates more efficient coordination. We created a dyad-level variable for analysis [Effective Adjustment rate = Effective Adjustment trials (Sub01 +Sub02)/(2 × Total trials)]. Furthermore, mean cooperation rate was defined as the percentage of “win” trials out of all experimental trials for each dyad [= win trials/ Sum trials].

We calculated the effective adjustment rate and the mean cooperation rate and then submitted these metrics to one-way ANOVAs with Group as the between-subjects factor. These metrics were also calculated for Block1 and Block2, respectively, and were submitted to one-way ANOVAs to further explore temporal dynamics, with Bonferroni correction for multiple comparisons. Additionally, for completeness, the increase in cooperative behavior (Block2-Block1) was also calculated to directly examine changes in cooperative behavior. One-sample *t*-tests with Bonferroni correction were used to analyze the increase in the effective adjustment rate and the mean cooperation rate for each emotion group.

### fNIRS data preprocessing

fNIRS data were preprocessed using the Homer2 software package and a customized MATLAB-based script in MATLAB 2021a (Mathworks Inc., Natick, MA, USA). Briefly, raw optical intensity signals were first converted into optical density (OD) and visually inspected to assess signal quality. Channels exhibiting detector saturation or poor optical coupling as marked by a lack of the heart beat frequency (∼1 Hz) in the signal’s power spectrum were removed (Pinti et al. 2017), resulting in 97.8% of channels being saved for further analysis. These channels with poor signal quality were replaced with the averaged neighboring channels (Kaiser et al. 2014; Zhang et al. 2021). Next, global physiological noises such as skin blood flow were removed via principal component analysis, with a 75% threshold of variance (Scholkmann et al. 2014). The signal time series were then screened and corrected for motion artifacts using a wavelet-based algorithm with an interquartile range of 0.5 (Molavi and Dumont 2012). A band-pass second-order Butterworth filter with cut-off frequencies of 0.01–0.8 Hz was applied to reduce slow drift and high-frequency noise. The filtered OD data were then converted into oxyhemoglobin (HbO) and deoxyhemoglobin (HbR) concentration changes based on the modified Beer–Lambert Law (Hiraoka et al. 1993, Hoshi 2007).

Consistent with previous fNIRS-based literature (e.g. Cui et al. 2012; Li et al. 2022), we primarily focused on HbO data due to its higher sensitivity to changes in regional cerebral blood flow and its superior signal-to-noise ratio (Hoshi 2007). Additionally, increases in HbO values are recorded as a consequence of neural activity and correspond to the blood oxygenation level-dependent signal measured by fMRI (Huppert et al. 2006, Kirilina et al. 2012).

### Assessment for cooperation-relatedIBS

We focused on the analysis of cooperation-related IBS between two individuals’ fNIRS data. The Wavelet Transform Coherence (WTC) algorithm was used to estimate the synchronous activity between the two HbO time series for each emotion group, each dyad, and each channel combination (CH) (Grinsted et al. 2004). WTC can reveal locally phase-locked behavior that may not be uncovered by traditional time series analysis, such as Pearson’s correlation (Grinsted et al. 2004). These data generated a 2D (time × frequency) matrix of coherence values. We focused on the 0.05–0.2 Hz frequency band (5–20s), as this range effectively captures the temporal structure of the task phases while encompassing frequencies observed in most previous similar studies (Cui et al. 2012, Tang et al. 2016; Li et al. 2022). Additionally, this frequency band excludes low-frequency fluctuations (below 0.01 Hz) and high-frequency physiological signals, such as cardiac pulsation (∼1 Hz) and respiration (∼0.3 Hz) (Pierro et al. 2014, Yücel et al. 2021). We then averaged the coherence values of this band during the Block1, Block2, and Total sessions for each cooperation task, respectively. Consistent with previous studies, the coherence values were converted into Fisher *z*-values to generate a normal distribution (Li et al. 2022, Zhang et al. 2021).

We assessed task-related significant channels in each emotion group using a nonparametric permutation approach based on phase-randomized surrogate data (Piazza et al. 2020, Yang et al. 2020). Previous studies have argued that the resting phase may not be an ideal baseline for IBS, as changes in IBS could reflect task-evoked alterations in the autonomic nervous system rather than neural activity (Scholkmann et al. 2022). Specifically, surrogate data for each channel timeseries were generated by performing a discrete Fourier transform, randomizing the phase of each Fourier component and then inverting the Fourier transformation. IBS was then recalculated using the surrogate data and averaged across dyads for each cooperative task and each channel. This procedure was repeated 2000 times to yield 2000 coherence value sets (null distribution) for each channel and each emotion group. The significance level was evaluated by comparing coherence values from the original dataset to those from the null distribution of the surrogate dataset. Multiple comparisons were performed across channels using the false-discovery rate (FDR; Benjamini and Hochberg 1995) (threshold *P*_FDR_ < .05). The significant IBS channels for each cooperative task within each emotional group are presented in Supplementary Figs S1 and S2.

#### PDG task IBS analyses.

Channels showing significant IBS in at least one emotional group during the PDG were submitted to one-way ANOVAs with Groups as the between-subjects factor, FDR was utilized to correct *P*-values for multiple comparisons. Additionally, to explore the temporal dynamics at the neural level, IBS in Block1 and Block2 were separately submitted to one-way ANOVAs with FDR correction. To directly examine dynamic changes of IBS, the increase in IBS (change of IBS = IBS_block2_—IBS_block1_) was quantified and submitted to a one-sample *t*-test. The resulting *P*-values using FDR correction for multiple comparisons across each retained channel and each emotional group. Next, to elucidate the mechanistic processes linking cooperative performance to neural signals, Pearson correlation coefficients were calculated to investigate the relationship between cooperative behaviors (CC, CD, and DD) and IBS from significant channels in Block1 and Block2, respectively. The resulting *P*-values were corrected using the FDR method for multiple comparisons.

### BPG task IBS analyses

We conducted parallel analyses in the BPG task. Specifically, channels exhibiting significant IBS in at least one emotional group during the BPG were submitted to one-way ANOVAs with Groups as the between-subjects factor, for Block1, Block2 and Total, for each retained channel, respectively. The FDR method was used to correct the resulting *P*-values. Then, the change in IBS was submitted to one-sample *t*-tests to directly examine the dynamic changes in IBS, with FDR correction for multiple comparisons. Additionally, we examined the relationship between neural signals and cooperation performance by calculating Pearson’s correlation coefficients between cooperative behaviors (Effective Adjustment rate, Mean Cooperation rate) and IBS from the significant channels in both Block1 and Block2, respectively.

#### IBS validation by pseudo-dyad analyses.

To confirm that the detected IBS was specific to real dyads interacting during the task rather than results of common experience, a validation approach was applied using the pseudo-dyad permutation procedure. Within each emotion group, original participant dyads were randomly assigned to new pseudo-dyads to recompute the IBS. This procedure was repeated 1000 times to generate a null distribution of pseudo-dyad IBS, which was then compared with the coherence values of real dyads in the corresponding channels for each condition. We further compared the observed group effect of real interacting dyads against the 1000 permutation (*F*-values) to examine whether real effect exceeded the upper 95% limits of the permutation null distributions. This procedure was constrained to the channels exhibiting significant effects of interest, significant levels (*P* < .05) were assessed by contrasting the coherence values or *F*-values from the original dyads with 1000 renditions of pseudo-dyads.

#### Exploratory analyses.

Finally, as an exploratory analysis, we sought to investigate the overlap and distinction of neural signals between two distinct modalities of cooperation (Zhang et al. 2024). Specifically, channels demonstrating significant IBS in at least one emotional group for each cooperation task were identified and retained. Then, we calculated the differences in IBS between the two cooperation tasks using paired-sample *t*-tests within those channels. The resulting *P*-values were corrected for multiple comparisons using the FDR method.

## Results

### Manipulation check of emotion induction

A linear mixed-effects model on the rating of ‘general positivity’ revealed a main effect of Round [*χ*^2^(2) = 74.55, *P* < .001], suggesting that participants experienced higher “pleasant” in Round 2 compared to the baseline and Round 1. There was also a main effect of Group [*χ*^2^(2) = 3.52, *P* = .033], with higher “pleasant” in the Gratitude and Joy compared to the Neutral. Additionally, a significant Round × Group interaction was found [*χ*^2^(4) = 24.55, *P* < .001], Post hoc analysis showed higher general positivity ratings in Round 2 (Gratitude: 6.67 ± 1.51; Joy: 6.09 ± 1.55) compared to Baseline (Gratitude: 4.81 ± 1.63; Joy: 4.32 ± 1.67) and Round 1 (Gratitude: 3.15 ± 1.09; Joy: 3.69 ± 1.29) in the Gratitude and Joy (all *Ps*_bonf_ < .01), but no significant differences among baseline, Round 1 and Round 2 in the Neutral (3.88 ± 1.64 vs 4.44 ± 1.56 vs 4.22 ± 1.48, *Ps*_bonf_ > .05). Furthermore, we assessed the the proportion of participants who “felt grateful to their partner” between groups using a Chi-square test. As expected, the results showed a larger proportion of “Yes” in the Gratitude (96.4%) compared to the Joy [16.7%; *χ*^2^(1) = 34.15, *P* < .001, *V* = 0.81] and the Neutral [6.7%; *χ*^2^(1) = 34.15, *P* < .001, *V* = 0.81], but no significant difference between the Joy and Neutral [*χ*^2^(1) < 0.001, *P* = .99, *V* < 0.001]. These results, which include all participants, confirm the successful manipulation of the specified emotional experiences. We excluded these dyads from subsequent analyses in which participants did not feel grateful toward their partner in gratitude and those who felt grateful to their partner in the joy and neutral. This procedure resulted in a final sample size of 78 dyads [29 in Gratitude, 24 in Joy and 25 in Neutral; aged 21.37 ± 2.14 years (mean ± s.d.)].

### Cooperative behavior and psychological measures in the PGD task

A two-way mixed-model ANOVA on the general outcomes (Total) showed a significant main effect of Type (*F*(2, 76) = 8.93, *P* = .001, *η*^2^partial = 0.263). Post hoc analysis with Bonferroni correction revealed that CC (0.36 ± 0.08) and CD (0.37 ± 0.07) were significantly higher than DD (0.27 ± 0.07). Notably, the Group × Type interaction effect was significant (*F*(4, 76) = 5.96, *P* = .004, *η*^2^partial = 0.191), indicating higher CC in the Gratitude (0.46 ± 0.05) compared to Joy (0.33 ± 0.06) and Neutral (0.27 ± 0.06). However, lower CD in the Gratitude (0.36 ± 0.06) compared to Neutral (0.43 ± 0.07), lower DD in the Gratitude (0.21 ± 0.05) compared to Joy (0.29 ± 0.08) and Neutral (0.30 ± 0.09).

**Figure 2.**
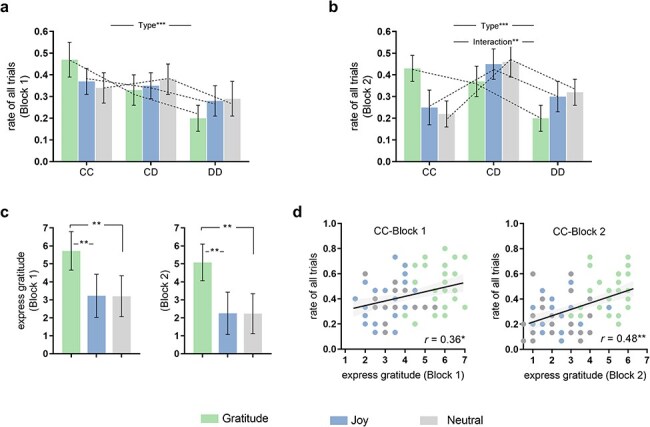
Results of behavioral and psychological measures in the PGD task. (a) General cooperation outcomes for the Block1. (b) General cooperation outcomes for the Block2. Results showed a significant type main effect was found in Block1 (a) and a significant type main effect and a group × type interaction effect in Block2 (b). (c) Psychological measures. Higher “express gratitude” in the Gratitude compared to the Joy and Neutral. (d) Correlation between “express gratitude” and CC in Block1 and Block2. *P < .05, **P < .01, ***P < .001, *n.s*.: not significant. Error bars indicate standard deviations.

Then, to further elucidate the temporal dynamics of cooperative behaviors, we repeated the above analyses for Block1 and Block2. As depicted in Fig. 2a, Block1 exhibited a significant main effect of Type (*F*(2, 76) = 9.89, *P*_bonf_ = .001, *η*^2^partial = 0.263) where CC (0.41 ± 0.08) and CD (0.34 ± 0.09) were significantly higher than DD (0.26 ± 0.07). However, no interaction effect was found (*F*(4, 77) = 3.43, *P*_bonf_ = .077, *η*^2^partial = 0.101). In Block2 (Fig. 2b), a significant Type main effect (*F*(2, 76) = 16.34, *P*_bonf_ = .001, *η*^2^partial = 0.395) revealed that CD (0.42 ± 0.07) was higher than CC (0.30 ± 0.08) and DD (0.28 ± 0.09). Additionally, the Group × Type interaction effect was significant (*F*(4, 77) = 12.36, *P*_bonf_ < .001, *η*^2^partial = 0.308), aligning with the Total findings, higher CC in the Gratitude (0.43 ± 0.06) compared to Joy (0.26 ± 0.08) and Neutral (0.20 ± 0.06), yet, lower CD and DD (CD: 0.36 ± 0.07; DD: 0.21 ± 0.06) compared to Joy (CD: 0.43 ± 0.07; DD: 0.47 ± 0.07) and Neutral (CD: 0.31 ± 0.07; DD: 0.33 ± 0.06). Subsequently, complementary temporal dynamic analyses by direct comparisons between Block1 and Block2 showed no significant differences in each type within the Gratitude (all *Ps*_bonf_ > 0.05, paired-sample *t*-tests with Bonferroni correction). However, we observed a significant decrease in CC (*t*(23) = -3.68, *P*_bonf_ = 0.016, *d* = -1.368; Neutral: *t*(24) = -3.74, *P*_bonf_ = 0.015, *d* = -1.456), alongside a significant increase in CD (Joy: *t*(23) = 3.06, *P*_bonf_ = 0.028, *d* = 1.200; Neutral: *t*(24) = 3.75, *P*_bonf_ = 0.014, *d* = 1.496) in the Joy and Neutral in Block2 compared to Block1. Additionally, a significant increase in DD was observed in the Joy (*t*(23) = 3.22, *P*_bonf_ = 0.021, *d* = 1.234).

One-way ANOVAs were performed on the five psychological measures at Block1 and Block2, respectively. As depicted in Fig. 2c, a significant group difference was observed in the rating of “express gratitude” at both Block1 (*F*(2, 76) = 20.67, *P*_bonf_ < .001, *η*^2^partial = 0.233) and Block2 (*F*(2, 76) = 34.24, *P*_bonf_ < .001, *η*^2^partial = 0.313). Post hoc comparisons with Bonferroni correction revealed higher “express gratitude” in the Gratitude compared to Joy and Neutral, at both Block1 (Gratitude vs Joy vs Neutral: 5.64 ± 1.46 vs 3.23 ± 1.79 vs 3.02 ± 1.84) and Block2 (5.12 ± 1.42 vs 2.84 ± 1.78 vs 2.77 ± 1.81). Notably, in alignment with the results of “express gratitude”, participants reported higher ratings of “trust the partner” in the Gratitude compared to Joy and Neutral (Block1: *F*(2, 76) = 18.22, *P*_bonf_ < .001, *η*^2^partial = 0.205; Block2: *F*(2, 76) = 26.14, *P*_bonf_ < .001, *η*^2^partial = 0.264). See Supplementary Table S2 and S3 for more supporting evidence. Additionally, we repeated above mixed-model ANOVAs while controlling for four other psychological contributors (“trust the partner,” “establish justice,” “to get money,” and “fulfill an obligation”) as covariates of noninterest. A similar pattern of results was obtained when including these variables as covariates (*Fs* > 6.08, *Ps* < .05), and no influence of covariates was found (all *Ps *> .05).

Moreover, Pearson’s correlations with Bonferroni correction were conducted between each of the five psychological measures and the general cooperation outcomes. The results demonstrated a significantly positively correlation between the rating of “express gratitude” and CC at both Block1 and Block2 (*r*_blcok1_ = 0.35, *P*_bonf_ = .016; *r*_blcok2_ = 0.49, *P*_bonf_ = .002) (Fig. 2d). Additionally, the rating of “trust the partner” was also significantly positively correlated with CC (*rs* > 0.38, *Ps*_bonf_ < .021, Supplementary Table S4). No significant correlations were found between the other three psychological measures and the general cooperative outcomes after Bonferroni correction (Supplementary Table S4).

In summary, these results suggest that participants tend to be grateful to others when they receive help from them, which facilitates trust and enhances cooperative interactions. Moreover, while both positive emotion groups yielded increased positivity, only the gratitude group predicted a socially inclusive attitude, characterized by participants’ reduced sensitivity to slight defects from their benefactors.

### Cooperative behavior performance in the BPG task

**Figure 3.**
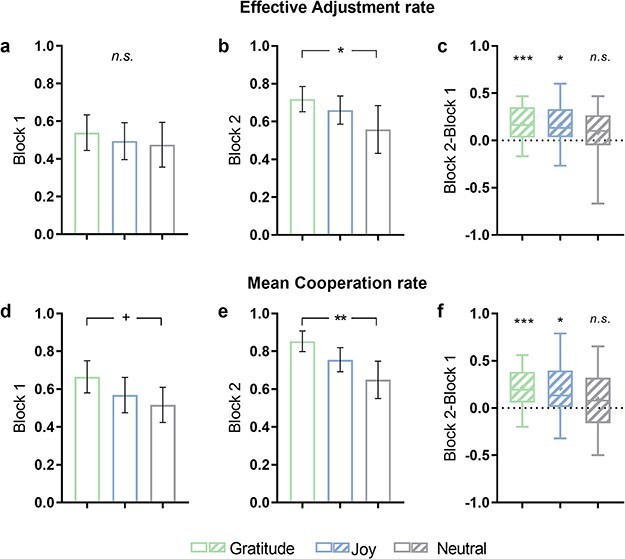
Behavioral results in the BPG task. (a–c) Effective adjustment rate for the Block1 (a), Block2 (b), and the difference between Blocks (Block2-Block1) (c). A significantly higher effective adjustment rate in the Gratitude than Neutral in the Block2 (b) while a marginally significant difference in Block1 (a). A significantly higher effective adjustment rate than zero was found in the Gratitude and Joy but not in the Neutral, for the difference between blocks c. (d–f) Mean cooperation rate for the Block1 (d), Block2 (e) and the difference between blocks (Block2-Block1) (f). Significant effects were rarely showed in Block2 (e), suggesting a higher mean cooperation rate in the Gratitude than Neutral. A significantly higher mean cooperation rate than zero was found in the Gratitude and Joy, but not in the Neutral, for the difference between blocks (f). *P < .05, **P < .01, ***P < .001, **+**: marginally significant, *n.s*.: not significant. Error bars indicate standard deviations.

One-way ANOVA on the effective adjustment rate indicated a significantly higher rate in the Gratitude (0.75 ± 0.11) compared to the Neutral (0.60 ± 0.15) (*F*(2, 76) = 5.58, *P* = .007, *η*^2^partial= 0.122). Then, one-way ANOVAs were respectively conducted for Block1 and Block2 to further explore temporal dynamics. The results revealed a marginally significant difference between groups at Block1 (*F*(2, 76) = 3.24, *P*_bonf_ = .063, *η*^2^partial = 0.058. Figure 3a) and a significant group effect at Block2 (*F*(2, 76) = 8.43, *P*_bonf_ = .001, *η*^2^partial = 0.168. Figure 3b). Post hoc comparisons at Block2 revealed a higher effective adjustment rate in the Gratitude (0.85 ± 0.10) compared to the Neutral (0.65 ± 0.20). Additionally, one-sample *t*-tests were conducted to directly examine the dynamic effect (i.e. Block2-Block1), revealing a significantly larger effective adjustment rate than zero in both the Gratitude (*t*(28) = 5.81, *P*_bonf_ < .001, *d* = 2.158) and the Joy (*t*(23) = 3.79, *P*_bonf_ = .004, *d* = 1.489), but not in the Neutral (*t*(24) = 1.52, *P*_bonf_ = .247, *d* = 0.589) (Fig. 3c).

Additionally, a parallel analysis was performed on the mean cooperation rate, revealing a significant group difference in Block2 (*F*(2, 76) = 3.76, *P*_bonf_ = .030, *η*^2^partial = 0.084, Fig. 3e), but not in Total (*F*(2, 76) = 2.41, *P*_bonf_ = .095, *η*^2^partial = 0.042) or Block1 (*F*(2, 76) = 0.43, *P*_bonf_ = .626, *η*^2^partial = 0.007, Fig. 3d) (one-way ANOVAs with Bonferroni correction). Post hoc comparisons in Block 2 showed a significantly higher mean cooperation rate in the Gratitude (0.72 ± 0.15) compared to the Neutral (0.59 ± 0.24). Additionally, one-sample *t*-tests with Bonferroni correction on the change of mean cooperation rate (Block2-Block1) showed a significantly larger increase in the mean cooperation rate than zero in both the Gratitude (*t*(28) = 4.54, *P*_bonf_ = .014, *d* = 1.682) and the Joy (*t*(23) = 2.82, *P*_bonf_ = .032, *d* = 1.106), but not in the Neutral (*t*(24) = 1.16, *P*_bonf_ = .264, *d* = 0.464) (Fig. 3f).

Finally, one-way ANOVAs were conducted on the post-task questionnaire, revealing higher ratings of ‘satisfaction with their own performance’ in the Gratitude and Joy groups compared to the Neutral (*F*(2, 76) = 4.64, *P*_bonf_ = .031, *η*^2^partial = 0.148). There was also a higher rating of ‘perceived cooperativeness’ in the Gratitude compared to the Joy and Neutral (*F*(2, 76) = 4.95, *P*_bonf_ = .016, *η*^2^partial = 0.188). Detailed information regarding the post-task questionnaire can be discovered in Supplementary Table S5. These findings offer evidence that feelings of gratitude and joy progressively promote cooperation in a costless cooperation situation. Moreover, this promoting effect is particularly pronounced in gratitude-induced cooperation as it associated with heightened satisfaction in interpersonal relationships.

### IBS result in the PDG task

**Figure 4.**
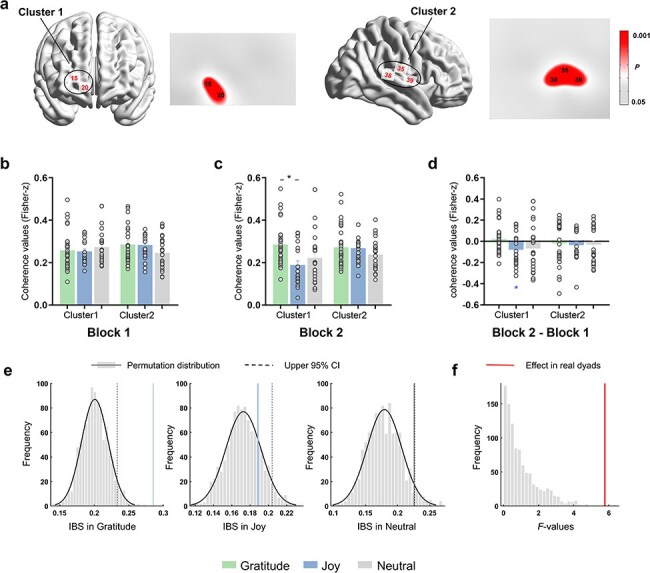
IBS results in the PDG task. (a) Using a nonparametric permutation approach, task-related significant channels were selected and clustered into two clusters. (b–d) IBS for each cluster with emotional group comparisons at the Block1 (b), Block2 (c), and the difference between Blocks (Block2-Block1) (d). Results showed a significant group difference at Cluster1 in Block2 (c), revealing larger IBS in the Gratitude compared to Joy. For the difference in IBS, there was a significant decrease in IBS in the Joy (d). *P < .05. Error bars indicate standard deviations. (e) The pseudo-dyad permutation results for each emotion group. Vertical solid lines indicate the positions of the true means of IBS for the original dyads relative to the distributions of the permuted data, and dotted lines indicate the upper 5% areas of the permutation distributions. (f) The group effect in IBS of the original dyads against the distribution of pseudo-dyad permuted *F*-values (*n* = 1000), exceeded the upper limits of the permutation distribution.

Based on a non-parametric permutation approach, task-related significant channels were selected and clustered into two clusters due to their spatial proximity (Cluster1: CH15 and CH20, roughly located at the right middle frontal gyrus; Cluster2: CH35, CH38, and CH39, roughly located at the right supramarginal gyrus). (Fig. 4a). Supplementary Fig. S1 provides detailed information on task-related significant channels for each emotion group. One-way ANOVAs on IBS for each cluster with FDR correction showed no significant difference in IBS between groups (Cluster1: *F*(2, 76) = 1.612, *P*_FDR_ = .298, *η*^2^partial = 0.081; Cluster2: *F*(2, 76) = 1.298, *P*_FDR_ = .377, *η*^2^partial = 0.082). One-way ANOVAs with FDR correction on IBS were then further conducted for Block1 and Block2, respectively. As seen in Fig. 4c, the results only exhibited a significant group difference in Cluster1 for Block2 (*F*(2, 76) = 5.773, *P*_FDR_ = .019, *η*^2^partial = 0.109). Post hoc comparisons revealed larger IBS in the Gratitude (0.29 ± 0.09) compared to Joy (0.19 ± 0.10). No significant effect of IBS was found for Total or Block1 (all *Ps*_FDR_ > .05). In addition, we observed a significant decrease in IBS in the Joy in terms of the difference of IBS (*t*(23) =  −2.82, *P*_FDR_ = 0.046, *d *= −1.139, one-sample *t*-test; Fig. 4d). We then conducted pseudo-dyad permutation tests to examine whether these detected channels showed increased IBS in real dyads compared to pseudo dyads. We constrained to the IBS that exhibited significant effects (Cluster1 for the Block2 in the PDG task). Validation results from the 1000 permutations for each emotion group were shown in Fig. 4e. IBS in original dyads was significantly higher than in pseudo-dyads in the gratitude (*P* < .001) and was marginally significantly higher in the neutral (*P* = .057), while the detected IBS in the joy was under the distribution (*P* = .18). Moreover, the observed effects of group in IBS were specific to the real dyads, as the *F*-value of the actual dyads exceeded the upper limits of the 95% areas of the permutation distribution (*P* < .001, Fig. 4f).

Next, Pearson correlations were applied to explore the relationships among IBS and cooperative performances (CC, CD, DD) across the Total, Block1 and Block2, respectively. Surprisingly, no evidence emerged supporting an association between IBS and cooperative performance, as no significant correlation were found (all *Ps*_FDR_ > .05, see Supplementary Table S6). Thus, it seems that the observed IBS may not be simply explained by individual measures of emotion-induced cooperation. As a supplementary analysis, we examined the direct differences in IBS among cooperation types (CC vs CD vs DD). The results demonstrated that the IBS of CC was significantly larger than that of CD and DD at Cluster1 (*F*(2, 76) = 4.29, *P*_FDR_ = .030, *η*^2^partial = 0.127, repeated-measures ANOVA). This finding indicates that the IBS at Cluster1 might play a role in predicting each other’s intentions and achieving mutual cooperation.

### IBS result in the BPG task

**Figure 5.**
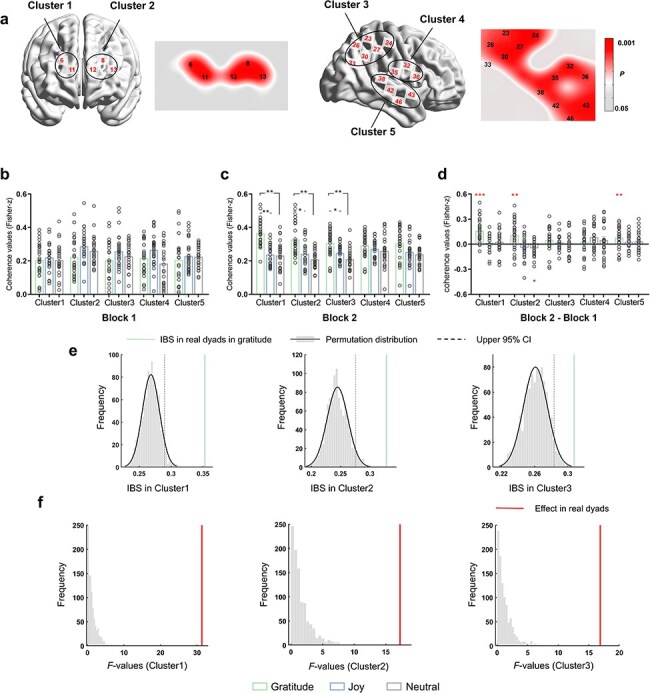
IBS results in the BPG task. (a) Using a nonparametric permutation approach, task-related significant channels were selected and clustered into five clusters. (b–d) IBS for each cluster with emotional group comparisons at the Block1 (b), Block2 (c) and the difference between Blocks (Block2-Block1) (d). Larger IBS was observed in the Gratitude compared to the Joy and Neutral at Cluster1, Cluster2 and Cluster3 in Block2 (c). For the difference in IBS, results showed a significant increase in IBS at Cluster1, Cluster2 and Cluster5 in the Gratitude, a significant decrease in IBS at Cluster2 in the Neutral (d, asterisk). *P < .05, **P < .01, ***P < .001, *n.s*. not significant. Error bars indicate standard deviations. (e) The pseudo-dyad permutation results for the three detected clusters in the gratitude group. Vertical solid lines indicate the positions of the true means of IBS for the original dyads relative to the distributions of the permuted data, and dotted lines indicate the upper 5% areas of the permutation distributions. (f) The group effect in IBS of the original dyads against the distribution of pseudo-dyad permutation *F*-values (*n* = 1000) for the three detected Clusters, all true *F*-values exceeded the upper limits of the permutation distributions.

The BPG task-related significant channels were identified using a non-parametric permutation approach and clustered into five clusters (Cluster1: CH6 and CH11, roughly located at the right middle frontal gyrus; Cluster2: CH8, CH12, and CH13, roughly located at the left middle frontal gyrus; Cluster3: CH23, CH24, CH26, CH27, CH30, and CH33, roughly located at the right sensorimotor cortex; Cluster4: CH32, CH35, and CH36, roughly located at the right supramarginal gyrus; Cluster5: CH38, CH42, CH43, and CH46. roughly located at the superior temporal gyrus) (see Fig. 5a). Supplementary Fig. S2 shows details about the task-related significant channels in each emotion group. Subsequently, one-way ANOVAs with FDR correction for each IBS cluster showed significant group differences in Cluster1 (*F*(2, 76) = 11.143, *P*_FDR_ < .001, *η*^2^partial = 0.224) and Cluster3 (*F*(2, 76) = 7.496, *P*_FDR_ < .01, *η*^2^partial = 0.128). Post hoc comparisons showed significantly larger IBS in the Gratitude (0.29 ± 0.05) compared to the Joy (0.23 ± 0.05) and Neutral (0.22 ± 0.06) in Cluster1. There was also a significantly larger IBS in the Gratitude (0.28 ± 0.05) than the Neutral (0.21 ± 0.06) in Cluster2.

Additionally, regarding the temporal dynamics of IBS, no significant clusters for Group were detected in Block1 (*Ps*_FDR_ > .05) (Fig. 5b). However, in Block2 (Fig. 5c), significant group differences emerged in Cluster1 (*F*(2, 76) = 31.347, *P*_FDR_ < .001. *η*^2^partial = 0.457), Cluster2 (*F*(2, 76) = 16.892, *P*_FDR_ < .001. *η*^2^partial = 0.279), and Cluster3 (*F*(2, 76) = 17.223, *P*_FDR_ < .001, *η*^2^partial = 0.298). Post hoc comparisons consistently revealed larger IBS in the Gratitude (Cluster1: 0.37 ± 0.07, Cluster2: 0.33 ± 0.09; Cluster3: 0.31 ± 0.08) compared to the Joy (0.23 ± 0.06; 0.24 ± 0.08; 0.25 ± 0.05) and Neutral (0.23 ± 0.08; 0.21 ± 0.05; 0.21 ± 0.06). No other effects were found. Furthermore, the change in IBS showed a significant increase in Cluster1 (*t*(28) = 6.60, *d* = 2.520), Cluster2 (*t*(28) = 3.56, *d* = 1.287) and Cluster5 (*t*(28) = 3.35, *d* = 1.243) in the Gratitude (*Ps*_FDR_ < .05, one-sample *t*-tests with FDR-correction. Figure 5d). Additionally, a significant decrease in IBS was observed in the Neutral at Cluster2 (*t*(24) =  −2.83, *P*_FDR_ = 0.015, *d* = −1.148). The pseudo-dyad permutation procedure was repeated for the IBS that exhibited significant effects in the BPG task (Cluster1, Cluster2, and Cluster3 for the Block2). Validation results from the 1000 permutations for each emotion group were shown in Fig. 5e and Supplementary Fig. S2. IBSs of original dyads in gratitude were significantly higher than those in the pseudo-dyads permutations at all detected clusters (*Ps* < .001, Fig. 5e). Additionally, all detected clusters of IBSs in original dyads in the joy were significantly higher than those in the permutations and marginally significantly higher IBSs in Cluster1 and Cluster2 in neutral (see Supplementary Fig. S3 for more details). Moreover, the observed group effect in IBS across all detected clusters were also specific to the real interacting dyad, as the *F*-values for the actual dyads exceeded the upper limits of the 95% area of the permutation distributions (*Ps* < .001, Fig. 5f).

Furthermore, Pearson correlations with FDR correction were employed to examine the relationships between IBS and cooperative performance at each cluster in the Total, Block1, and Block2, respectively. Significant positive correlations were found between the Effective Adjustment rate and IBS at Cluster1 in the Total (*r* = 0.47, *P*_FDR_ = .002) and Block2 (*r* = 0.46, *P*_FDR_ = .005). However, no significant correlations were found between the Mean Cooperation rate and IBS (all *Ps*_FDR_ > .05). Please refer to Supplementary Table S7 for detailed descriptions.

### Neural signals (IBS) overlap and distinction between the two types of cooperation tasks

An exploratory examination was conducted to determine the overlap and distinctions between the two modalities of cooperation in this study. Channels exhibiting significant task-related IBS were retained based on the nonparametric permutation approach. We found that the right middle frontal gyrus and the right supramarginal gyrus showed significant task-related IBS across both the PDG and BPG tasks. This suggests that IBS in these regions may serve a fundamental role in the cognitive processes underlying social cooperative interactions. Additionally, neural distinctions between the PDG and BPG tasks revealed significantly larger IBS in the BPG compared to the PDG at CH8, CH24, CH27, and CH42 (all *ts* < −2.54, *Ps*_FDR_ < .041, paired-sample *t*-test with FDR correction). These channels are roughly located in the left middle frontal, sensorimotor cortex, and right superior temporal gyrus. More details are available in Supplementary Table S8.

## Discussion

Gratitude has been conceptualized as a response to benefiting from another’s intentional actions. This study investigates the dynamic impact of gratitude on various modalities of cooperative behaviors and the underlying interpersonal neural mechanisms, yielding several critical findings: participants exhibited superior cooperative behaviors in both the PDG and BPG tasks when experiencing gratitude compared to joy and neutral states. The temporal dynamics of cooperative behaviors further elucidate that gratitude dynamically facilitates cooperation by boosting inclusiveness of slight defects from benefactors and heightening action coordination. Consequently, IBS was higher in the gratitude group than in the joy or neutral groups, and it increased dynamically over time (blocks) during the BPG task. Therefore, our results provide evidence that gratitude induces widespread social cooperation and increases IBS over time. Our work contributes to the understanding of gratitude-induced cooperation in several important ways, as discussed below.

### Gratitude induces widely cooperative interactions

Gratitude, a social–moral emotion, has been associated with numerous positive effects, including enhanced self-satisfaction and improved mental and physical well-being (DeSteno et al. 2010, Bartlett et al. 2012). Our findings in the PDG task align with these studies, demonstrating that experiencing gratitude facilitates the recognition of kindness and increases the inclination to engage in cooperative behavior in costly situations (Balconi and Fronda, 2021, Bartlett et al. 2012, Tang et al. 2016). Traditionally, cooperation favoring communal profit at the expense of self-interest has been thought to stem from strategic control aimed at mitigating emotional responses centered on immediate resource acquisition. Thus, our study adds evidence that gratitude can facilitate cognitive inhibition of self-centered impulses and devalue immediate self-interest (Bartlett et al. 2012, Zhu et al. 2021). We also provide insights into the underlying psychological mechanisms underlying gratitude-induced cooperation. Self-reports in the gratitude group showed “express gratitude” to their partner, rather than merely reciprocating or fulfilling obligations as evidenced by “establish justice” and “fulfill an obligation”. Moreover, we found a positive correlation between CC and “trust the partner.” Indeed, previous studies have provided empirical evidence that trust is foundation for cooperative relationships (Kausel and Connolly 2014), and gratitude has been shown to influence monetary decisions in trusting social interactions (Lount 2010, Drążkowski et al. 2017). Therefore, our results support the view that a grateful recipient is likely to be a reliable and trustworthy cooperative partner, which is essential for initiating fruitful interdependent cooperation. Future research endeavors are encouraged to investigate this aspect.

To our knowledge, this study is the first to assess gratitude-induced cooperation using the BPG task. Our results demonstrate significantly higher effective adjustment rates and mean cooperation rates in gratitude-induced cooperative behaviors. As mentioned in our previous study (Li et al. 2022), effective adjustment reflects a stronger motivation for autonomous modulation of concrete actions, indicating that improved cooperation in gratitude dyads may be attributed to increased cooperative efficiency and strategic interactions between individuals. Moreover, according to interdependence theory (Hertel et al. 2000, Zhang et al. 2024), individuals need to comprehend and predict the actions of others, whereby psychology and behavior work together to accomplish a common goal in the BPG task. Therefore, higher cooperative behaviors in gratitude dyads reflect a stronger sense of ‘joint agency’ (Shiraishi and Shimada 2021). Our self-report findings also support this hypothesis, showing higher evaluations of performance and relationship satisfaction in the gratitude dyads.

Importantly, this study demonstrates that the effect of gratitude cannot be simply attributed to experiencing a positive emotional state, as the joy emotion did not promote cooperation. This differentiation states with the same valence is crucial for advancing our understanding of gratitude’s role in guiding cooperation. Notably, we found no statistically significant differences between gratitude and joy in the BPG, despite numerically higher effective adjustment rate and mean cooperation rate in the gratitude group. This finding may not be surprising, given that an earlier study from our group showed a promoting effect of positive emotion in the BPG task (Li et al. 2022). Therefore, the current study suggests that the boost effect on cooperative behaviors may not be unique to gratitude and further research is warranted to specifically clarify this possibility.

### Gratitude dynamically tracks cooperative interactions

The current study provides groundbreaking insights into how gratitude dynamically influences cooperative interactions. Specifically, the PDG task revealed no significant differences among the emotion groups in Block1. However, after receiving feedback from Block1, participants’ in the joy and neutral groups showed a marked decrease in cooperation but an increase in defection in Block2. These findings align with previous research and can be explained by fairness and reciprocity norms, which suggest that individuals treat others in the same way they are treated (Condon and DeSteno 2011, Jahng et al. 2017). When participants were aware of that their partner had increased defections, they were more inclined to choose defection subsequently. Interestingly, this trend was more pronounced in the joy dyads, indicating a heightened sense of self-threat and stronger competitive impulses when facing an uncooperative partner while experiencing joy (Balconi and Vanutelli, 2017).

Importantly, yet, we found no significant change in cooperative behaviors in the gratitude in PDG. Namely, a slight defect from benefactors does not provoke retaliation. Our study supports the notion that gratitude acts as a ‘‘moral barometer’’, drawing one’s attention to kind acts and serving as a moral motivator to encourage continued behavior that fosters relationships (McCullough et al. 2001, Bartlett et al. 2012). In other words, gratitude does not characterize a tit-for-tat reciprocity but rather inhibits short-term selfish motivations, fostering decisions and actions centered on communal benefit (van Kleef and Lelieveld, 2022). Alternatively, grateful individuals may be insensitive to provocation and thus more likely to accommodate rather than resist a benefactor’s destructive actions (Algoe 2012, Gordon et al. 2013). In summary, our findings provide evidence that cooperation stemming from sincere intentions, rather than precise calculations of cooperation costs, signals a high-quality and long-term cooperative relationship that responds to gratitude.

Additionally, we observed dynamic increases in the effective adjustment rates and mean cooperation rates across blocks in the BPG task for the gratitude and joy groups. This finding suggests that positive emotional experiences contribute to better coordination of concrete actions with others for successful cooperation (Li et al. 2022). Importantly, this facilitating effect cannot be attributed to a simple learning process, as increased cooperative behaviors were not observed in the neutral group. Moreover, the effective adjustment rate showed emotion differences in both blocks, while the mean cooperation rate showed such differences only in Block2. This finding is partly supported by our previous study, which suggests that the effective adjustment rate represents an effort to work together psychologically and behaviorally, and might be more sensitive and earlier in predicting cooperative quality (Li et al. 2022). It is noteworthy that, despite numerically larger cooperation in gratitude than in joy, no statistically significant difference was found between these two positive emotion groups. However, higher self-reported perceived cooperativeness and recognition of their partner’s contributions in the gratitude compared to higher self-reported contributions of themselves in the joy suggest that gratitude is not merely a general positive state. Grateful individuals appear to be more attuned to recognizing and affirming their partner’s contributions during interactions.

### Gratitude induces dynamic larger cooperation-related IBS

At the neural level, our study enhances the understanding of the across-brain neural mechanisms involved in gratitude-induced cooperation. Specifically, our fNIRS results observe higher cooperation-related IBS in the left and right middle frontal gyrus and the sensorimotor cortex. Numerous fNIRS-based studies have demonstrated that the middle frontal gyrus is involved in adaptive behaviors, perspective taking, and predicting relevant information about others in various complex social interactions (Li et al. 2022, Shi et al. 2014, Liu et al. 2021). Our study further confirms the functional role of the middle frontal gyrus in social interaction behaviors. Furthermore, IBS in the sensorimotor cortex suggests a sensorimotor “tuning in” or “syncing” between individuals during cooperation, which facilitates understanding other’s intentions and actions (Dumas et al. 2020, Gamliel et al. 2021). Additionally, while some social cognition and mentalizing regions, such as the supramarginal gyrus and the superior temporal gyrus, were also observed in the present research, no significant differences in these regions were found between emotion groups. One explanation is that these brain regions possibly play a universal role in the cognitive process of interactions and cooperation (Xie et al. 2020), requiring further confirmatory research.

Noticeably, the temporal dynamics of IBS further underscore the functional role of these regions in the cognitive processes underlying social interactions and cooperation, aligning with our behavioral findings that significant results were predominantly focused in Block2. Moreover, although no significant correlation was found between IBS and cooperative behaviors in the PDG, complementary difference analysis showed that higher IBS in the right middle frontal gyrus was associated with more mutual cooperative behaviors (CC) in the PDG task, thereby supporting the IBS-behavior association from a novel perspective. Interestingly, our results showed an earlier IBS effect in the BPG task, with larger IBS in the right middle frontal gyrus and the sensorimotor cortex in Block1 in the gratitude group. These findings demonstrate that IBS may enable earlier and more sensitive identification of cooperative performance compared to behavioral patterns (Zhou et al. 2021).

In further exploration, we examined the overlap and distinction of neural signals between the two cooperation tasks. Our findings suggest that the right middle frontal gyrus and the right supramarginal gyrus may serve a common role in guiding and establishing social cooperation, despite variability across channels. Importantly, our results showed larger IBS in the BPG compared to the PDG in the left middle frontal, sensorimotor cortex, and the right superior temporal gyrus. Several explanations are possible. One explanation is that the BPG task involves joint attention and shared goals, requiring individuals to coordinate their attention and actions towards a common objective. Therefore, the higher IBS observed in the BPG may act as cognitive control for coordinating behaviors, such as adjusting one’s button-press actions in accordance with their partner, thereby contributing to cooperation (Li et al. 2022). Alternatively, the distinct setup and social processing of the two tasks may account for the difference. In the PDG, participants have difficulty inferring their partner’s decision while planning their own. This uncertainty about other’s intentions may harm neural synchronization across individuals (Wang et al. 2015, Tang et al. 2016). In summary, these findings reflect differences in neural representations underlying the social cognition inherent in the two types of cooperative behaviors.

### Limitations and future directions

It is plausible that this study has not exhausted all possibilities. We call for future studies to our explorations. First, only female dyads were tested in accordance with previous practice and recommendations (Baker et al. 2016, Cheng et al. 2015; Liu et al., 2023). Future research involving male and mixed-gender dyadic interactions is necessary to extend the validity of the present findings. Besides, the generalizability of our findings is limited to young adults. Future studies could investigate gratitude emotion and neural signatures underlying cooperation dynamics through a developmental lens (Vaish and Savell 2022). Moreover, as gratitude is deemed to be shaped by culture, we believe that cultural replications would be an interesting addition to this body of research (Curry et al. 2019). Second, as the antithesis of gratitude, ingratitude is likely to elicit negative responses from benefactors. It would be interesting for future research to investigate the dynamic influence of ingratitude on cooperative interactions and the associated neural mechanisms. Moreover, our choice of cooperation modalities might not fully represent all cooperative behaviors; more experimental work is needed to systematically understand how gratitude fosters cooperative behaviors. Importantly, we limited cooperation to restricted computer-based games instead of contextualized speaking to real or business situations. Despite this setting ensuring that our findings would not be attributed to idiosyncratic features of contextualized cooperation (Kong and Belkin 2019), we extremely encourage researchers to contextualize these findings by examining multiple realistic tasks outside of lab-created settings. These endeavors promise to deepen our knowledge of gratitude’s role in establishing and strengthening social connections.

## Conclusion

In summary, this study furnishes evidence supporting the notion that gratitude facilitates widespread social cooperation and enhances inter-brain synchronization among individuals. These findings deepen our comprehension of the social functions of gratitude, thereby bolstering the emergence and success of large-scale cooperative societies.

## Supplementary Material

nsaf023_Supp
