# Supplementary material for: Gratitude enhances widespread dynamic cooperation and inter-brain synchronization in females
Source: Soc Cogn Affect Neurosci. 2025 Mar 17;20(1):nsaf023. doi: 10.1093/scan/nsaf023 (PMC12426888; doi:10.1093/scan/nsaf023)
Supplement: nsaf023_Supp [file nsaf023_supp.zip › scan-24-141-File007.docx]

***Supplementary material***

**Gratitude Enhances Widespread Dynamic Cooperation and Inter-brain Synchronization in females**

Yangzhuo Li^1^, Xinyu Cheng^6^, Wanqiu, Na^4^, Junlong Luo^1,2#^, Xianchun Li^3,5#^

^1^ School of Psychology, Shanghai Normal University, Shanghai, China

^2^ Lab for Educational Big Data and Policymaking, Ministry of Education, Shanghai Normal University, Shanghai, China

^3^ Shanghai Key Laboratory of Mental Health and Psychological Crisis Intervention, Affiliated Mental Health Center (ECNU), School of Psychology and Cognitive Science, East China Normal University, Shanghai, China

^4^ Department of psychogeriatrics, Third People's Hospital of Huzhou, Huzhou, China.

^5^ Institute of Wisdom in China, East China Normal University, Shanghai, China.

^6^ Shanghai Key Laboratory of Psychotic Disorders, Shanghai Mental Health Center, Shanghai Jiaotong University School of Medicine, Shanghai, China

**Supplementary Table 1.** Demographic, Pre-task emotion scale among Groups by One-way ANOVA.

| Measurement | Mean ± SD | | | *F* | *P*-corr |
| --- | --- | --- | --- | --- | --- |
|  | Gratitude | Joy | Neutral |  |  |
| **• Demographic information** | | | | | |
| Age (year) | 22.23(2.35) | 22.43(3.02) | 21.98(2.71) | 0.765 | 0.677 |
| Education year | 17.89(1.74) | 16.76(2.06) | 17.44(2.55) | 0.378 | 0.862 |
| **• Pre-task emotion scale** | | | | | |
| positive affect (PA) | 2.83(0.12) | 3.18(0.13) | 3.22(0.19) | 1.422 | 0.554 |
| negative affect (NA) | 1.12(0.14) | 1.10(0.15) | 1.53(0.26) | 0.697 | 0.698 |

Positive affect (PA: active, alert, attentive, determined, enthusiastic, excited, inspired, interested, proud, and strong) and Negative affect (NA: afraid, ashamed, distressed, guilty, hostile, irritable, jittery, nervous, scared, and upset), on a 5-point scale (1= very slightly or not at all, 5 = extremely). Scores were computed for PA and NA for each emotional condition by averaging the items from the PANAS subscales.

**Supplementary Table 2.** Self-report Psychological Measures in the PDG task among Groups by One-way ANOVA.

| Measures | Gratitude | Joy | Neutral | ANOVA | | |
| --- | --- | --- | --- | --- | --- | --- |
|  | *M* (*SD*) | *M* (*SD*) | *M* (*SD*) | *F* | *P-*corr | *η^2^_p_* |
| **Block 1** |  |  |  |  |  |  |
| express gratitude | 5.64 (1.46) | 3.23 (1.79) | 3.02 (1.83) | 20.676 | **< 0.001** | 0.217 |
| trust the partner | 4.79 (2.01) | 2.74 (1.68) | 2.44 (1.87) | 18.225 | **< 0.001** | 0.204 |
| to get money | 4.18 (1.47) | 4.69 (1.76) | 4.62 (2.03) | 3.758 | 0.217 | 0.073 |
| establish justice | 4.33 (1.46) | 4.21 (1.82) | 4.13 (1.91) | 2.167 | 0.924 | 0.019 |
| fulfill an obligation | 3.45 (1.81) | 4.26 (1.68) | 4.24 (1.81) | 4.562 | 0.083 | 0.068 |
| **Block 2** |  |  |  |  |  |  |
| express gratitude | 5.12 (1.42) | 2.84 (1.78) | 2.77 (1.81) | 34.344 | **< 0.001** | 0.318 |
| trust the partner | 4.26 (2.02) | 1.74 (1.64) | 1.46 (1.83) | 26.142 | **< 0.001** | 0.279 |
| to get money | 4.28 (1.47) | 5.69 (1.78) | 5.43 (1.93) | 7.092 | **0.023** | 0.104 |
| establish justice | 4.36 (1.45) | 5.21 (1.84) | 5.07 (2.01) | 5.461 | **0.038** | 0.093 |
| fulfill an obligation | 4.34 (1.80) | 3.86 (1.65) | 4.11 (1.83) | 2.364 | 0.448 | 0.025 |

**Supplementary Table 3.** Psychological Measures among Block1 and Block2 in the PDG task by paired-sample *t* test.

| Block 1 vs Block 2 | Gratitude | Joy | Neutral |
| --- | --- | --- | --- |
|  | *t* (*P-*corr) | *t* (*P-*corr) | *t* (*P-*corr) |
| express gratitude | 0.186 (0.938) | 1.432 (0.206) | 1.328 (0.216) |
| trust the partner | 0.745 (0.914) | **2.734 (0.043)** | 1.287 (0.265) |
| to get money | 0.086 (1.017) | **-3.271 (0.032)** | **-3.047 (0.038)** |
| establish justice | 0.047 (1.009) | **-3.042 (0.037)** | **-3.112 (0.032)** |
| fulfill an obligation | -0.230 (0.949) | 2.017 (0.174) | 1.879 (0.484) |

**Supplementary Table 4.** Pearson correlation between each of the five psychological measures and cooperation general outcomes in the PDG task.

| Psychological Measures | CC | CD | DD |
| --- | --- | --- | --- |
|  | *r* (*P-*corr) | *r* (*P-*corr) | *r* (*P-*corr) |
| **Block 1** |  |  |  |
| express gratitude | **0.35 (0.018)** | 0.11 (0.656) | 0.09 (1.00) |
| trust the partner | **0.38 (0.019)** | 0.26 (0.213) | -0.16 (0.622) |
| to get money | -0.13 (0.439) | 0.16 (0.587) | 0.15 (0.574) |
| establish justice | 0.21 (0.237) | -0.20 (0.256) | 0.23 (0.289) |
| fulfill an obligation | -0.14 (0.486) | -0.05 (1.00) | 0.19 (0.453) |
| **Block 2** |  |  |  |
| express gratitude | **0.49 (0.012)** | -0.12 (0.656) | -0.06 (1.00) |
| trust the partner | **0.37 (0.025)** | 0.22 (0.343) | -0.19 (0.549) |
| to get money | 0.18 (0.347) | 0.27 (0.288) | 0.25 (0.176) |
| establish justice | 0.29 (0.219) | 0.25 (0.259) | 0.23 (0.369) |
| fulfill an obligation | -0.14 (0.565) | -0.15 (0.698) | -0.17 (0.537) |

**Supplementary Table 5.** Post-task questionnaire in the BPG task among Groups by One-way ANOVA.

|  | Gratitude | Joy | Neutral | ANOVA | | |
| --- | --- | --- | --- | --- | --- | --- |
| Measures | *M* (*SD*) | *M* (*SD*) | *M* (*SD*) | *F* | *P-*corr | *η^2^_p_* |
| Satisfaction with their own performance | 2.41 (0.63) | 2.38 (0.74) | 1.97 (0.67) | 4.213 | **0.036** | 0.145 |
| Satisfaction for their partner performance | 2.26 (0.70) | 2.71 (0.55) | 2.33 (0.73) | 3.031 | 0.066 | 0.104 |
| Perceived cooperativeness | 2.64 (0.69) | 2.23 (0.77) | 2.00 (1.23) | 5.068 | **0.014** | 0.189 |


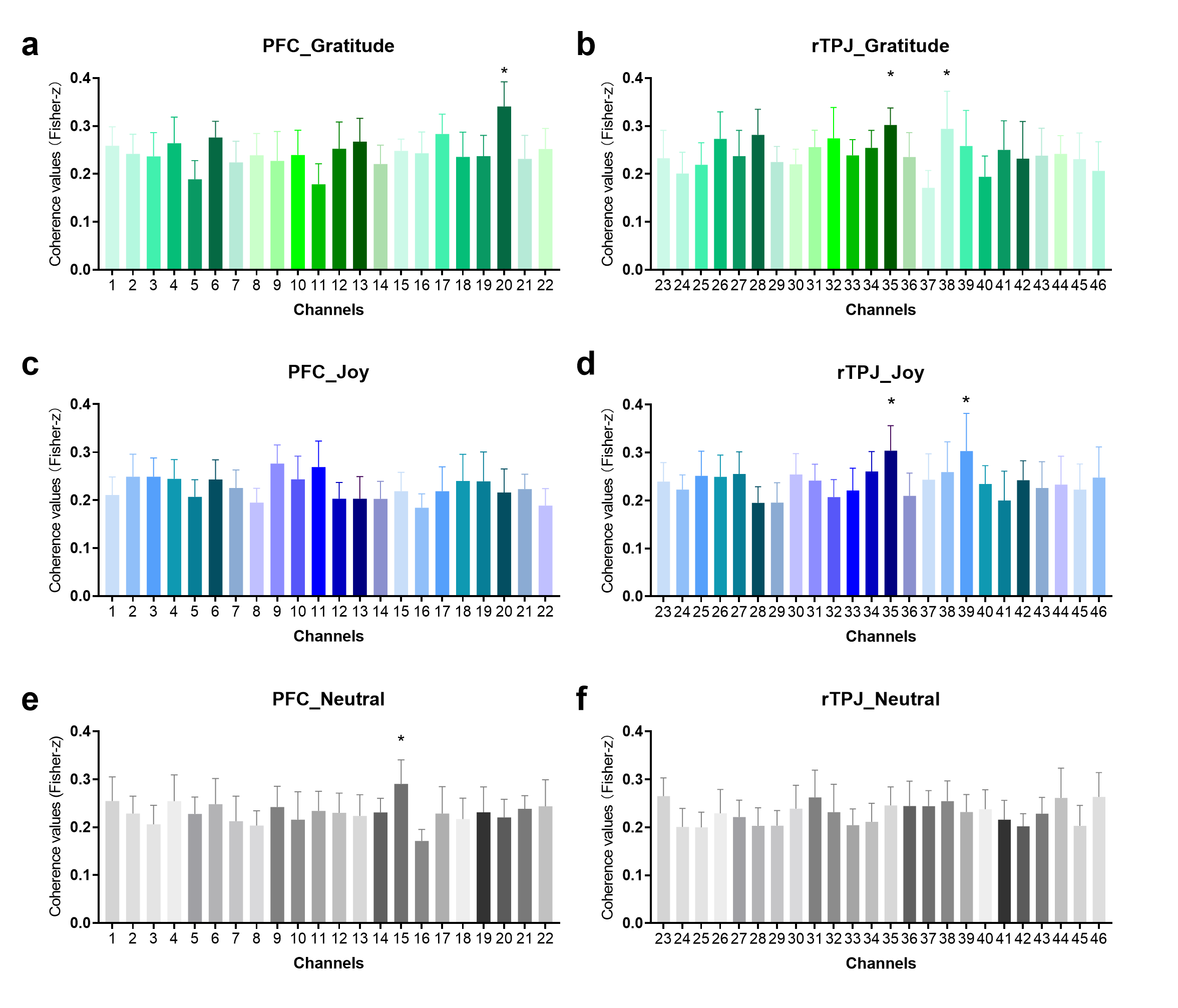


**Supplementary Figure 1.** Task-related significant channels in the PDG task using non-parametric permutation approach. channel 20, 35, and 38 were selected in the Gratitude; channel 35 and 39 were selected in the Joy; channel 15 was selected in the Neutral (*ps* < 0.05 after FDR correction).


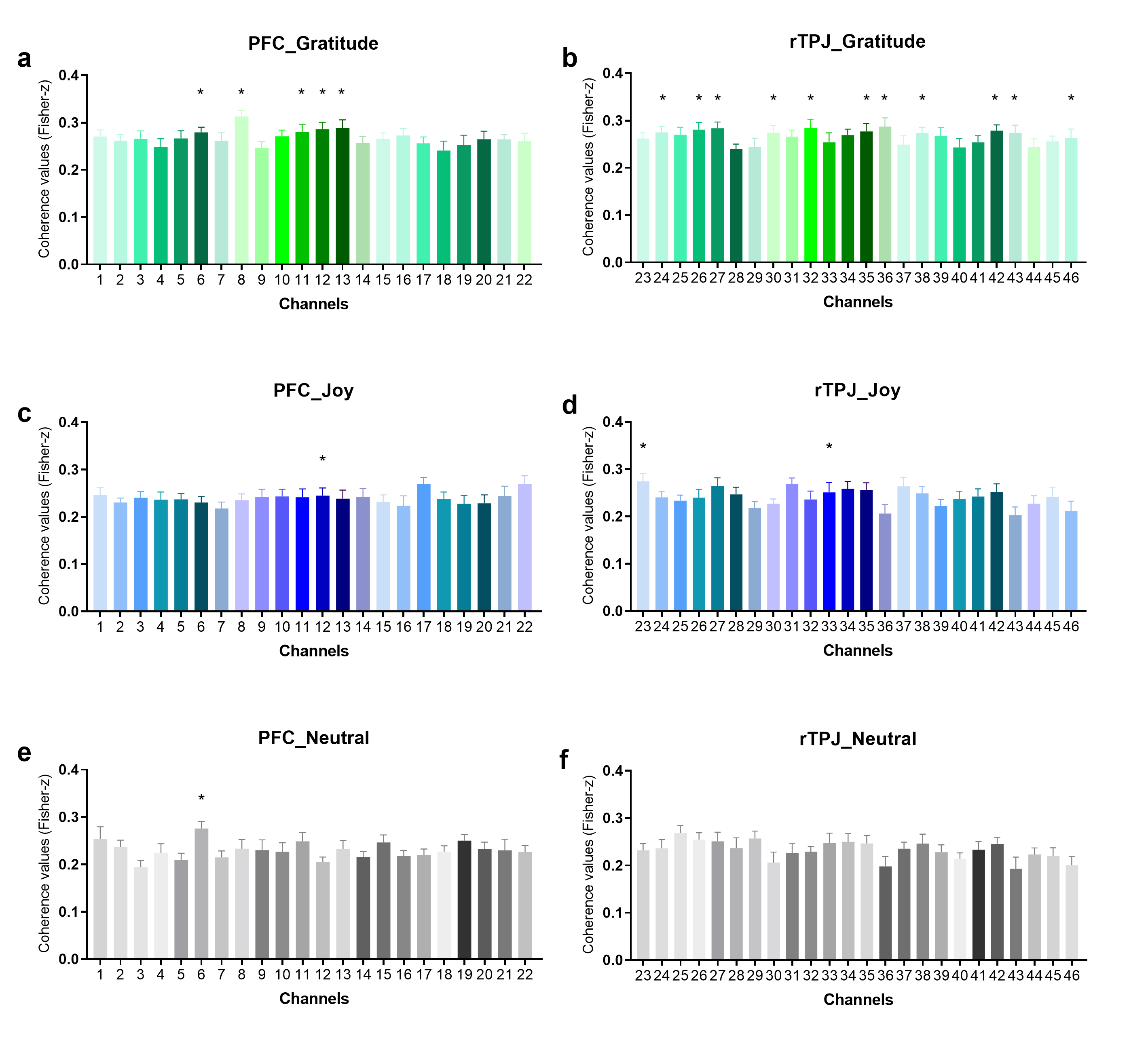


**Supplementary Figure 2.** Task-related significant channels in the BPG task using non-parametric permutation approach. channel 6, 8, 11, 12, 13, 24, 26, 27, 30, 32, 35, 36, 38, 42, 43, and 46 were selected in the Gratitude; channel 12, 23, and 33 were selected in the Joy; channel 6 was selected in the Neutral (*ps* < 0.05 after FDR correction).


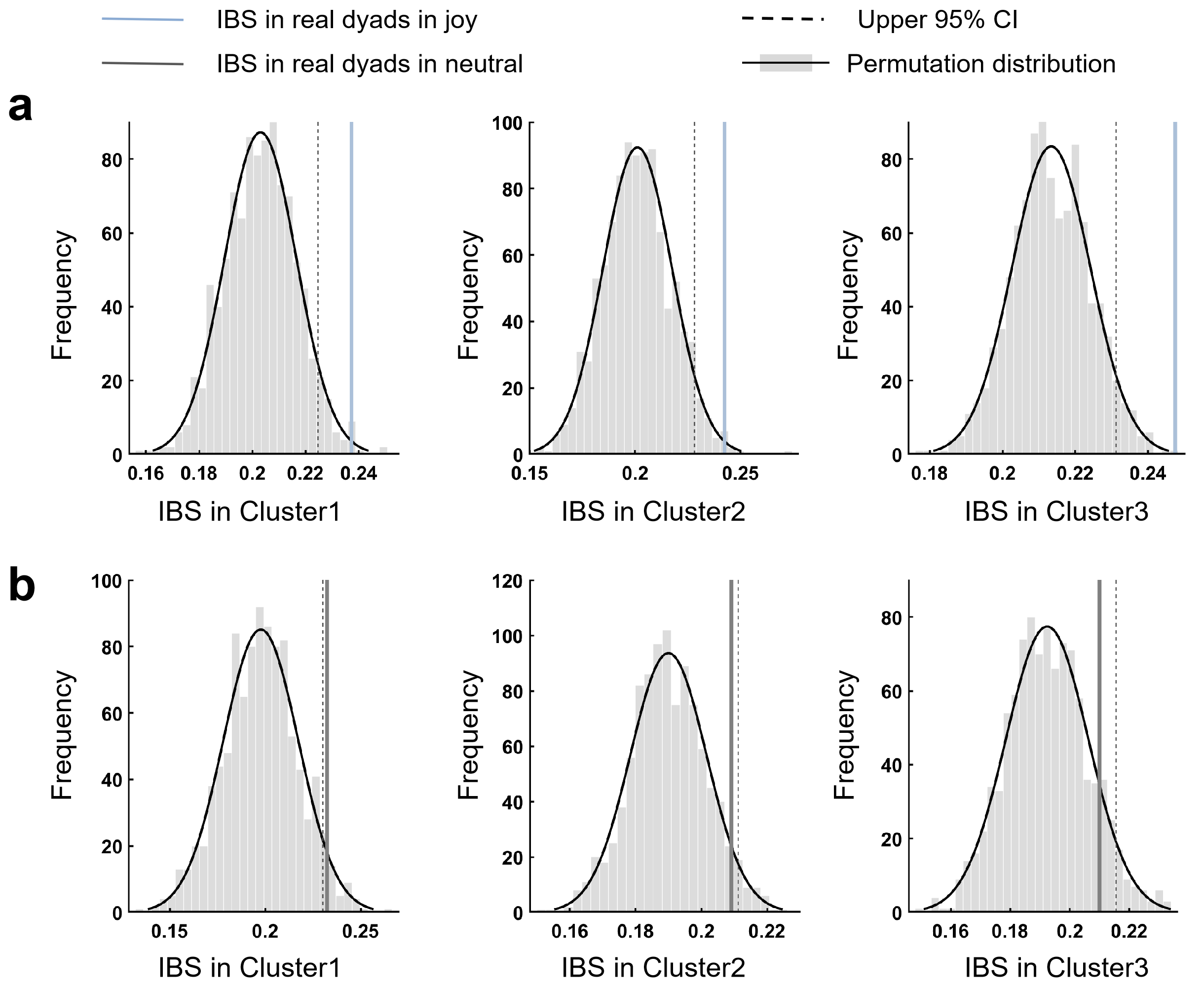


**Supplementary Figure 3. validation tests in joy and neutral groups in the BDP task. (a)** All detected clusters of IBSs of original dyads in the joy were significantly higher than in pseudo-dyads permutations (Cluster1 permutation: 95% CI: 0.157 0.224, Cluster2 permutation: 95% CI: 0.154, 0.28; Cluster3 permutation: 95% CI: 0.176, 0.231; *Ps* < 0.001). **(b)** in the neutral group, marginally significantly higher IBS in Cluster1 (permutation: 95% CI: 0.136 0.232) and Cluster2 permutation: 95% CI: 0.151 0.209), but IBS in the Cluster3 was under the pseudo-dyads distribution (permutation: 95% CI: 0.149 0.214, *P* = 0.138).

**Supplementary Table 6.** Pearson correlation between IBS and cooperation general outcomes in the PDG task.

|  | Cluster 1  *r* (*P-*corr) | | | Cluster 2  *r* (*P-*corr) | | |
| --- | --- | --- | --- | --- | --- | --- |
|  | Total | Block 1 | Block 2 | Total | Block 1 | Block 2 |
| CC | 0.14 (0.337) | 0.13  (0.369) | 0.21  (0.098) | 0.17  (0.316) | 0.14  (0.419) | 0.18  (0.212) |
| CD | -0.09  (0.846) | 0.10  (0.737) | 0.13  (0.414) | 0.09  (0.706) | 0.07  (0.855) | 0.10  (0.655) |
| DD | 0.21  (0.132) | 0.19  (0.294) | 0.14  (0.327) | 0.13  (0.528) | 0.06  (1.00) | 0.14  (0.406) |

**Supplementary Table 7.** Pearson correlation between IBS and cooperative behaviors in the BPG task.

|  | Effective Adjustment rate  *r* (*P-*corr) | | | Mean Cooperation rate  *r* (*P-*corr) | | |
| --- | --- | --- | --- | --- | --- | --- |
|  | Total | Block 1 | Block 2 | Total | Block 1 | Block 2 |
| Cluster 1 | **0.42 (0.007)** | 0.06 (0.819) | **0.46**  **(0.001)** | 0.12  (0.343) | 0.08  (0.764) | 0.17  (0.216) |
| Cluster 2 | 0.26 (0.067) | 0.01  (1.00) | 0.17  (0.326) | -0.06  (0.698) | 0.05  (0.946) | 0.09  (0.706) |
| Cluster 3 | 0.17  (0.226) | 0.09 (0.835) | 0.22  (0.124) | 0.09  (0.526) | -0.03  (1.00) | 0.17  (0.214) |
| Cluster 4 | 0.11 (0.406) | -0.02 (1.00) | 0.19  (0.285) | 0.20  (0.17) | -0.02  (1.00) | 0.16  (0.314) |
| Cluster 5 | 0.24  (0.086) | 0.05  (0.865) | 0.21  (0.106) | 0.19  (0.18) | 0.09  (0.842) | 0.06  (0.964) |

**Supplementary Table 8.** IBS among PDG task and BPG task by paired-sample *t* test.

|  | PDG | BPG | *t* | *P* | *P-*corr |
| --- | --- | --- | --- | --- | --- |
| Channels | *M* (*SD*) | *M* (*SD*) | － | － | － |
| 1 | 0.241(0.04) | 0.258(0.07) | － | － | － |
| 2 | 0.240(0.05) | 0.244(0.05) | － | － | － |
| 3 | 0.231(0.06) | 0.237(0.07) | － | － | － |
| 4 | 0.254(0.06) | 0.238(0.06) | － | － | － |
| 5 | 0.208(0.07) | 0.240(0.06) | － | － | － |
| 6 | 0.256(0.05) | 0.262(0.07) | － | － | － |
| 7 | 0.221(0.08) | 0.234(0.07) | － | － | － |
| **8** | 0.212(0.06) | 0.264(0.07) | **-4.322** | **< 0.001** | **0.009** |
| 9 | 0.248(0.06) | 0.240(0.07) | － | － | － |
| 10 | 0.233(0.07) | 0.249(0.07) | － | － | － |
| 11 | 0.227(0.07) | 0.259(0.07) | － | － | － |
| 12 | 0.229(0.05) | 0.249(0.07) | － | － | － |
| 13 | 0.231(0.08) | 0.256(0.04) | － | － | － |
| 14 | 0.218(0.05) | 0.240(0.07) | － | － | － |
| 15 | 0.252(0.07) | 0.249(0.06) | 0.256 | 0.794 | 0.827 |
| 16 | 0.199(0.07) | 0.241(0.06) | － | － | － |
| 17 | 0.243(0.06) | 0.250(0.07) | － | － | － |
| 18 | 0.231(0.06) | 0.236(0.07) | － | － | － |
| 19 | 0.236(0.07) | 0.244(0.07) | － | － | － |
| 20 | 0.259(0.07) | 0.244(0.07) | 1.355 | 0.181 | 0.333 |
| 21 | 0.231(0.05) | 0.248(0.06) | － | － | － |
| 22 | 0.228(0.07) | 0.254(0.07) | － | － | － |
| 23 | 0.246(0.05) | 0.257(0.06) | -0.880 | 0.383 | 0.568 |
| 24 | 0.208(0.06) | 0.252(0.06) | **-3.113** | **0.004** | **0.032** |
| 25 | 0.223(0.05) | 0.258(0.06) | － | － | － |
| 26 | 0.251(0.08) | 0.260(0.05) | -0.658 | 0.421 | 0.580 |
| 27 | 0.238(0.06) | 0.268(0.07) | **-2.518** | **0.025** | **0.043** |
| 28 | 0.227(0.07) | 0.241(0.06) | － | － | － |
| 29 | 0.208(0.07) | 0.239(0.06) | － | － | － |
| 30 | 0.238(0.05) | 0.239(0.06) | -0.009 | 0.993 | 0.993 |
| 31 | 0.253(0.06) | 0.255(0.07) | － | － | － |
| 32 | 0.237(0.05) | 0.253(0.07) | -1.355 | 0.181 | 0.333 |
| 33 | 0.221(0.07) | 0.251(0.07) | -2.214 | 0.025 | 0.093 |
| 34 | 0.242(0.05) | 0.260(0.08) | － | － | － |
| 35 | 0.284(0.07) | 0.261(0.08) | 1.617 | 0.112 | 0.291 |
| 36 | 0.230(0.07) | 0.235(0.07) | -0.325 | 0.746 | 0.817 |
| 37 | 0.219(0.05) | 0.250(0.07) | － | － | － |
| 38 | 0.269(0.05) | 0.258(0.07) | 0.883 | 0.383 | 0.568 |
| 39 | 0.264(0.06) | 0.241(0.06) | 1.711 | 0.093 | 0.267 |
| 40 | 0.222(0.06) | 0.233(0.05) | － | － | － |
| 41 | 0.222(0.05) | 0.244(0.06) | － | － | － |
| **42** | 0.225(0.05) | 0.260(0.07) | **-3.214** | **0.014** | **0.039** |
| 43 | 0.231(0.08) | 0.227(0.05) | 0.294 | 0.77 | 0.824 |
| 44 | 0.245(0.07) | 0.232(0.06) | － | － | － |
| 45 | 0.219(0.07) | 0.241(0.06) | － | － | － |
| 46 | 0.239(0.05) | 0.228(0.08) | 0.798 | 0.429 | 0.580 |

Note: Channels showing significant IBS in at least one emotional group for each cooperative task were retained.

**Supplementary Table 9.** The coordinates in MNI space and corresponding

neuroanatomical labels for channels

| Channel | MNI coordinate | | | AAL | | Brodman (Chris rorden’s MRIcro) | |
| --- | --- | --- | --- | --- | --- | --- | --- |
|  | x | y | z | Area | Percentage | Area | Percentage |
| **prefrontal** | | | | | | | |
| 1 | -36 | 63 | -7 | Frontal_Mid_Orb_L | 1.00 | 10 - Frontopolar area | 0.59 |
| 2 | -13 | 71 | -3 | Frontal_Mid_Orb_L | 0.56 | 11 - Orbitofrontal area | 0.77 |
| 3 | 15 | 71 | -3 | Frontal_Sup_Orb_R | 0.83 | 11 - Orbitofrontal area | 0.62 |
| 4 | 38 | 63 | -7 | Frontal_Mid_Orb_R | 0.96 | 10 - Frontopolar area | 0.62 |
| 5 | -45 | 53 | 1 | Frontal_Mid_L | 0.52 | 46 - Dorsolateral prefrontal cortex | 1.00 |
| 6 | -24 | 68 | 8 | Frontal_Sup_L | 0.96 | 10 - Frontopolar area | 0.97 |
| 7 | 2 | 68 | 9 | Frontal_Sup_Medial_L | 0.58 | 10 - Frontopolar area | 1.00 |
| 8 | 27 | 68 | 8 | Frontal_Sup_R | 1.00 | 10 - Frontopolar area | 0.93 |
| 9 | 47 | 53 | 2 | Frontal_Mid_R | 0.59 | 46 - Dorsolateral prefrontal cortex | 1.00 |
| 10 | -35 | 58 | 19 | Frontal_Mid_L | 1.00 | 46 - Dorsolateral prefrontal cortex | 0.92 |
| 11 | -13 | 67 | 22 | Frontal_Sup_L | 0.86 | 10 - Frontopolar area | 1.00 |
| 12 | 14 | 68 | 23 | Frontal_Sup_R | 0.74 | 10 - Frontopolar area | 1.00 |
| 13 | 38 | 59 | 18 | Frontal_Mid_R | 0.89 | 46 - Dorsolateral prefrontal cortex | 0.79 |
| 14 | -45 | 42 | 27 | Frontal_Mid_L | 0.89 | 45 - pars triangularis Broca's area | 0.92 |
| 15 | -23 | 56 | 33 | Frontal_Sup_L | 0.58 | 46 - Dorsolateral prefrontal cortex | 0.65 |
| 16 | 2 | 59 | 34 | Frontal_Sup_Medial_L | 0.74 | 10 - Frontopolar area | 0.53 |
| 17 | 26 | 57 | 33 | Frontal_Sup_R | 0.57 | 46 - Dorsolateral prefrontal cortex | 0.50 |
| 18 | 47 | 42 | 28 | Frontal_Mid_R | 0.89 | 45 - pars triangularis Broca's area | 0.94 |
| 19 | -35 | 40 | 42 | Frontal_Mid_L | 0.92 | 9 - Dorsolateral prefrontal cortex | 0.91 |
| 20 | -11 | 50 | 45 | Frontal_Sup_L | 0.68 | 9 - Dorsolateral prefrontal cortex | 1.00 |
| 21 | 13 | 50 | 46 | Frontal_Sup_Medial_R | 0.51 | 9 - Dorsolateral prefrontal cortex | 1.00 |
| 22 | 36 | 40 | 42 | Frontal_Mid_R | 1.00 | 9 - Dorsolateral prefrontal cortex | 0.97 |
| right temporo-parietal | | | | | | | |
| 23 | 23 | -56 | 74 | Parietal_Sup_R | 0.99 | 7 - Somatosensory Association Cortex | 0.73 |
| 24 | 36 | -33 | 72 | Postcentral_R | 0.82 | 4 - Primary Motor Cortex | 0.51 |
| 25 | 44 | -12 | 67 | Precentral_R | 0.86 | 4 - Primary Motor Cortex | 0.51 |
| 26 | 23 | -69 | 67 | Parietal_Sup_R | 0.99 | 7 - Somatosensory Association Cortex | 1 |
| 27 | 40 | -51 | 67 | Parietal_Sup_R | 0.78 | 7 - Somatosensory Association Cortex | 0.49 |
| 28 | 51 | -26 | 64 | Postcentral_R | 0.75 | 3 - Primary Somatosensory Cortex | 0.52 |
| 29 | 54 | -5 | 55 | Precentral_R | 0.49 | 6 - Pre-Motor and Supplementary Motor Cortex | 0.73 |
| 30 | 41 | -64 | 58 | Angular_R | 0.41 | 7 - Somatosensory Association Cortex | 0.59 |
| 31 | 57 | -43 | 56 | Parietal_Inf_R | 0.83 | 40 - Supramarginal gyrus part of Wernicke's area | 0.89 |
| 32 | 62 | -22 | 51 | SupraMarginal_R | 0.47 | 1 - Primary Somatosensory Cortex | 0.5 |
| 33 | 37 | -79 | 46 | Angular_R | 0.33 | 7 - Somatosensory Association Cortex | 0.50- |
| 34 | 55 | -58 | 50 | Parietal_Inf_R | 0.65 | 39 - Angular gyrus | 0.56 |
| 35 | 66 | -35 | 46 | SupraMarginal_R | 0.81 | 40 - Supramarginal gyrus part of Wernicke's area | 0.84 |
| 36 | 67 | -12 | 38 | Postcentral_R | 0.7 | 43 - Subcentral area | 0.34 |
| 37 | 50 | -74 | 39 | Angular_R | 0.85 | 39 - Angular gyrus | 0.86 |
| 38 | 64 | -51 | 38 | SupraMarginal_R | 0.38 | 40 - Supramarginal gyrus part of Wernicke's area | 0.75 |
| 39 | 70 | -28 | 33 | SupraMarginal_R | 1 | 2 - Primary Somatosensory Cortex | 0.62 |
| 40 | 42 | -87 | 24 | Occipital_Mid_R | 1 | 19 - V3 | 0.95 |
| 41 | 59 | -66 | 27 | Angular_R | 0.53 | 39 - Angular gyrus | 0.87 |
| 42 | 70 | -42 | 22 | Temporal_Sup_R | 0.63 | 22 - Superior Temporal Gyrus | 0.69 |
| 43 | 71 | -19 | 13 | Temporal_Sup_R | 0.73 | 22 - Superior Temporal Gyrus | 0.86 |
| 44 | 51 | -82 | 13 | Occipital_Mid_R | 0.78 | 19 - V3 | 0.86 |
| 45 | 65 | -59 | 11 | Temporal_Mid_R | 0.96 | 37 - Fusiform gyrus | 0.64 |
| 46 | 73 | -35 | 4 | Temporal_Mid_R | 0.65 | 22 - Superior Temporal Gyrus | 0.58 |
